# Differential Expression of Dopamine D5 Receptors across Neuronal Subtypes in Macaque Frontal Eye Field

**DOI:** 10.3389/fncir.2018.00012

**Published:** 2018-02-12

**Authors:** Adrienne Mueller, Steven B. Shepard, Tirin Moore

**Affiliations:** ^1^Department of Neurobiology, Stanford University, Stanford, CA, United States; ^2^Howard Hughes Medical Institute (HHMI), Stanford University, Stanford, CA, United States

**Keywords:** D5R, macaque, FEF, pyramidal neuron, parvalbumin, calbindin, calretinin, somatostatin

## Abstract

Dopamine signaling in the prefrontal cortex (PFC) is important for cognitive functions, yet very little is known about the expression of the D5 class of dopamine receptors (D5Rs) in this region. To address this, we co-stained for D5Rs, pyramidal neurons (neurogranin+), putative long-range projection pyramidal neurons (SMI-32+), and several classes of inhibitory interneuron (parvalbumin+, calbindin+, calretinin+, somatostatin+) within the frontal eye field (FEF): an area within the PFC involved in the control of visual spatial attention. We then quantified the co-expression of D5Rs with markers of different cell types across different layers of the FEF. We show that: (1) D5Rs are more prevalent on pyramidal neurons than on inhibitory interneurons. (2) D5Rs are disproportionately expressed on putative long-range projecting pyramidal neurons. The disproportionately high expression of D5Rs on long-range projecting pyramidals, compared to interneurons, was particularly pronounced in layers II–III. Together these results indicate that the engagement of D5R-dependent mechanisms in the FEF varies depending on cell type and cortical layer, and suggests that non-locally projecting neurons contribute disproportionately to functions involving the D5R subtype.

## Introduction

Dopamine signaling in the prefrontal cortex (PFC) is related to numerous cognitive functions including reward processing (Arias-Carrión et al., 2010), working memory (Puig et al., 2014) and attention (Noudoost and Moore, 2011a). Pharmacological manipulation of D1-family dopamine receptors (D1 receptors and D5 receptors) in one region of macaque PFC has been shown to modulate working-memory-related signals (Williams and Goldman-Rakic, 1995; Sawaguchi, 2001). Further, application of a D1/5R antagonist in another region of macaque PFC, the frontal eye field (FEF), elicits changes in the visual responses of neurons in posterior visual cortex that closely resemble those observed during attention (Noudoost and Moore, 2011a).

Although the distribution of D1Rs in the PFC has been well-examined, only two recent studies (Bordelon-Glausier et al., 2008; Glausier et al., 2009) have examined the expression of D5Rs in detail. However, in both cases the examination of the prevalence of dopamine D5 receptors on different cell classifications was mainly restricted to layer III of macaque dorsolateral prefrontal cortex (dlPFC, Area 9). Additionally, other previous studies that examined D5R expression in primates identified pyramidal neurons only morphologically (Bergson et al., 1995a,b; Ciliax et al., 2000), which limits quantification. Therefore, there has yet to be a comprehensive description of the expression of D5Rs across common cell classifications across all layers within PFC. An examination of D5R expression on different cell types, including subpopulations of pyramidal neurons and different interneuron classes, is informative because previous work has established that these different neuronal classes perform distinct roles in the cortical microcircuit (Kubota, 2014; Harris and Shepherd, 2015).

The FEF occupies a unique position within PFC as it provides an interface between the PFC and posterior visual cortex, as well as oculomotor output structures. Long-range pyramidal neurons in layers II–III of the FEF project to extrastriate visual cortical areas (Schall et al., 1995; Stanton et al., 1995; Anderson et al., 2011), whereas long-range pyramidal neurons in layer V project to the superior colliculus and to saccade generating structures in the pons (Segraves and Goldberg, 1987; Schall, 1997; Pouget et al., 2009). It has also been shown (Condé et al., 1994; Pouget et al., 2009) that calbindin+ and calretinin+ neurons are found predominantly in superficial layers of the FEF, whereas parvalbumin+ neurons are predominantly found in intermediate layers (Gabbott and Bacon, 1996; Disney and Aoki, 2008; Pouget et al., 2009; Zaitsev et al., 2009). Parvalbumin, calbindin, calretinin and somatostatin are markers for unique subpopulations of interneurons (DeFelipe, 1997; Gonchar and Burkhalter, 1997; Gonchar et al., 2007; Mascagni et al., 2009). Parvalbumin neurons predominantly synapse onto pyramidal neurons (DeFelipe, 1997) whereas calbindin and calretinin neurons primarily target other inhibitory neurons (Meskenaite, 1997; Gonchar and Burkhalter, 1999; Staiger et al., 2004; Wang et al., 2004). Somatostatin neurons, in turn, appear to powerfully inhibit pyramidal and inhibitory neurons alike (Fino and Yuste, 2011; Pala and Petersen, 2015; Urban-Ciecko and Barth, 2016). To address the potential role of D5R in a cortical microcircuit, we examined the distribution of D5Rs across different cell types and across different layers of the FEF, and asked whether the relative distributions (across different cell types) differed between layers.

## Materials and Methods

We performed immunofluorescence stains on sections from four male rhesus macaque brains. The animals were all adults at the time of perfusion. All experimental procedures were in accordance with NIH Guide for the Care and Use of Laboratory Animals, the Society for Neuroscience Guidelines and Policies, and the recommendations of the Stanford University Animal Care and Use Committee. The protocol was approved by the Stanford University Administrative Panel on Laboratory Animal Care.

### Fixation

The monkeys were anesthetized to the surgical plane with isofluorane then initially perfused with 0.25–0.5 liters serological saline at high pressure. After this they were perfused with 4 liters of 3.5%–4% paraformaldehyde in 0.1 M phosphate buffer: 2 liters at high pressure over 2–3 min, 2 liters at low pressure for an hour. Finally, they were perfused with 1 liter each of 10%, 20%, and 30% sucrose solutions at high pressure for cryoprotection. After perfusion, the brain rested in a 30% sucrose phosphate buffered solution for 7–10 days. We then used a freezing microtome to cut 20 μm coronal sections of the PFC. Slices were stored in phosphate buffered saline (0.1 M) until stained and imaged. Each of the animals had previously been used for electrophysiological experiments, but we did not include tissue that exhibited recording track damage in our analysis.

### Immunofluorescence

We co-stained sections with an antibody to the D5 dopamine receptor (αD5R) and different neuronal markers (see Table 1). We performed two controls to verify the specifity of the D5R antibody from Alomone Labs (ADR-005, see Supplementary Figure S1). Our working solution for antibody dilutions and washes was 0.1 M phosphate buffer contained 5% donkey serum (Millipore, S30–100ML) as a blocking agent. For approximately one third of our sections, our working solution also 0.5% Triton X-100 which is thought to improve permeability. We found no difference in staining with the inclusion of Triton X-100 in our working solution and ultimately discontinued using it. Sections were initially washed three times then exposed to the working solution (blocking buffer) for 60–90 min. Then the sections were washed three times again before being exposed to the primary antibodies at room temperature overnight. The following day the sections were then washed three times and exposed to an appropriate secondary antibody for 2 h at room temperature. We used donkey-anti-mouse, donkey-anti-goat, donkey-anti-rabbit or goat-anti-rat Alexafluor antibodies in 488, 568 or 647 wavelengths (ThermoFisher Scientific). The sections were then washed between 6 and 10 times further and exposed to 10 mM cupric sulfate in acetate solution to quench lipofuscin particle fluorescence (Schnell et al., 1999). Finally the sections were mounted on slides with DAPI-enriched fluoromount mounting medium (Vector Laboratories, Vectashield, H-1200). DAPI stains DNA and so is a label for all cells.

**Table 1:** Information on primary antibodies used for immunofluorescence.

Target	Description	Dilution	Product Information
D5R	Dopamine D5 receptor	1:200	Rabbit Polyclonal; ADR-005; Alomone Labs
Neuronal Nuclei N	All neurons	1:1000	Mouse Monoclonal; MAB377; Millipore
Neurogranin	Pyramidal neurons	1:200	Mouse Monoclonal; sc-514922; Santa Cruz Biotechnology
SMI-32	Putative long-range projection neurons	1:1000	Mouse Monoclonal; NE1023; Millipore
Parvalbumin	Inhibitory interneuron class	1:1000	Mouse Monoclonal; p3088; Sigma
Calbindin	Inhibitory interneuron class	1:1000	Mouse monoclonal; CB300; Swant Inc.
Calretinin	Inhibitory interneuron class	1:1000	Mouse Monoclonal; 6B3; Swant Inc.
Somatostatin	Inhibitory interneuron class	1:400	Rat Monoclonal; MAB354; Millipore

### Imaging

We identified the FEF as the rostral bank of the arcuate sulcus, posterior to the principle sulcus (Moschovakis et al., 2004; Percheron et al., 2015) and performed tile scans of continuous areas of cortex (pial surface to white matter) for both regions. Sections were imaged with a Leica TCS SP2 AOBS confocal microscope, using a 20× objective and were analyzed using ImageJ. We used estimated optical section thicknesses between 1.8 μm and 2.5 μm. We collected confocal Z-stacks spanning the 20 μm section depth and collapsed images across the Z-dimension for counting and illustration. Laser power, gain and offset were optimized at the beginning of an imaging session and not adjusted thereafter. Images were taken using sequential line scans with the different laser wavelength to reduce bleed-through.

### Quantification

We manually counted neurons that expressed either D5R, a neuronal marker, or both, on each image for each animal. Although cortical neurons are approximately 5–20 μm in diameter and rarely are physically abutting, it is theoretically possible that collapsing across the Z-dimension could hide neurons that are completely overlapping along the Z-axis. Our counts are therefore only estimates of the true number of neurons within an area. For some estimates, counts were pooled to generate a measure of the proportion of inhibitory neurons in general. We then averaged counts across all four animals. In some cases, we quantified expression for multiple sections for a single animal. In these circumstances, we first averaged within animal counts before averaging across animals. Proportions for co-expression were calculated from these across-animal averages. We estimated the number of neurons expressing a particular dopamine receptor, neuronal marker, or both across cortex by identifying their positions along the pia/white-matter axis. We identified the different cortical layers by visual inspection as follows: Layer I was identified as a region with very few cell bodies. Layer IV, which exists as a very narrow strip in macaque FEF (Huerta et al., 1986; Moschovakis et al., 2004; Percheron et al., 2015), was identified as a thin band with very small, tightly-packed cells. Layer II–III was therefore the region in between layers I and IV. Layer V was identified by the presence of large neurons with pyramidal cell morphology. Layer VI was therefore the region between layer V and the predominantly neuron-free white matter.

We made statistical comparisons between groups using chi-squared tests, or Fisher exact tests, and corrected for multiple comparisons using the Bonferonni method. The majority of comparisons were two-by-two: D5R presence and absence on two different cell types; resulting in a degree of freedom of one. In the case of across-layer comparisons for a single cell type, there were three degrees of freedom.

## Results

### Similar Expression of D5Rs across Different Layers of the FEF

**Figure 1:**
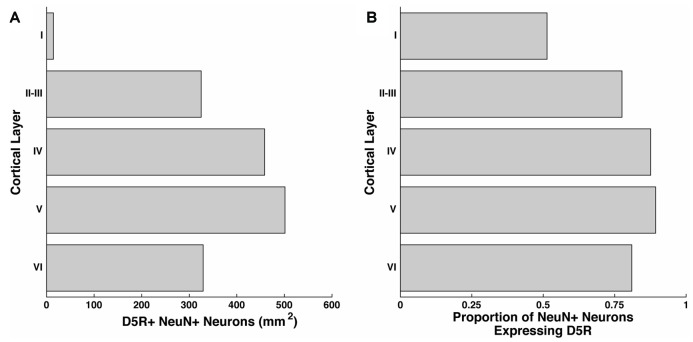
Distribution of D5Rs across cortical layers. **(A)** Number of NeuN+ neurons that co-express D5R for cortical layers I, II–III, IV, V and VI per mm^2^. **(B)** Proportion of NeuN+ neurons that express D5Rs for cortical layers I, II–III, IV, V and VI.

We examined the number of neurons per mm^2^ of D5R-expressing neurons in layers I, II–III, IV, V and VI of the FEF by co-staining for D5R and a pan-neuronal protein (NeuN). The NeuN antibody recognizes a neuron-specific nuclear protein, present in all cortical neurons of vertebrates (Mullen et al., 1992). We counted the number of neurons expressing D5Rs per mm^2^ of our 20 μm thick sections, collapsed across the Z-dimension. We found the highest proportion of D5R-expressing neurons in layers IV and V (458.4/mm^2^ and 501.2/mm^2^ respectively, Figure 1A). We identified somewhat lower densities of D5R+ cells in layers II–III (325.0/mm^2^) and VI (329.1/mm^2^). As expected, there was a low proportion of D5R+ neurons in layer I (14.5/mm^2^), which has very few neurons in general. We then examined the proportion of NeuN+ neurons expressing D5Rs (Figure 1B). We found that in contrast to the overall proportion of D5R+ NeuN+ neurons, a very similar proportion of neurons expressed D5Rs across all layers except layer I. Excluding layer I, there was no significant difference in the proportion of D5R+ neurons across layers (*p* = 0.26).

### Differential Expression of D5R in Different Cell Types

**Figure 2:**
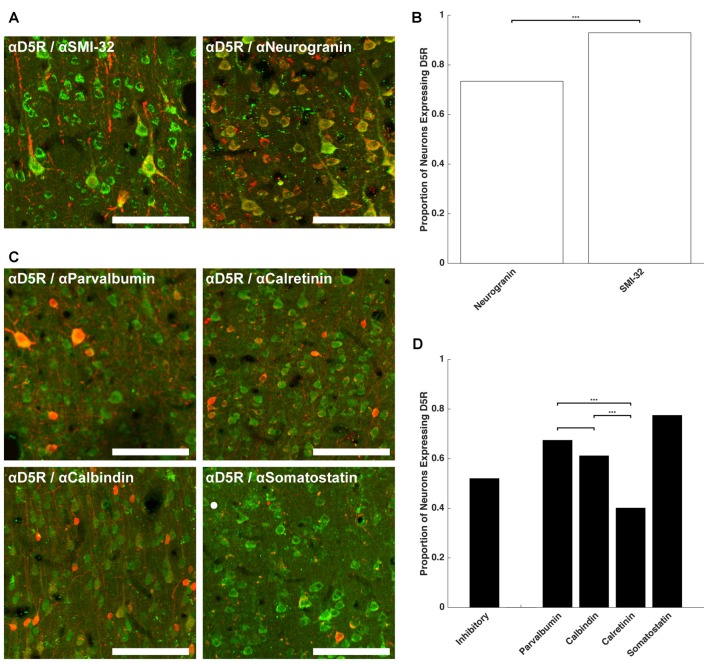
D5R expression on different cell types. **(A)** Co-expression of D5Rs (green) and SMI-32+ putative long-range projection pyramidal neurons (red; left) and co-expression of D5Rs with a general pyramidal neuron stain (neurogranin, red; right). **(B)** Proportion of frontal eye field (FEF) SMI-32+ and Neurogranin+ neurons that express D5Rs. **(C)** Expression of D5Rs (green) among different interneuron subtypes (red): parvalbumin+ (top left), calretinin+ (top right), calbindin+ (bottom left), somatostatin+ (bottom right). **(D)** Proportion of inhibitory interneurons that express D5Rs. Statistical comparisons were made between parvalbumin+, calbindin+ and calretinin+ neurons. Somatostatin+ neurons were excluded because they were too sparse to be used in statistical comparisons. For all panels: scale bar is equal to 100 μm and *** denotes significance at the level *p* ≤ 0.001.

Having established that a similar proportion of neurons express D5Rs across different layers (barring layer I), we next examined whether D5R expression differed across different cell types. We co-stained for D5Rs and two markers of pyramidal neurons and four markers of different classes of inhibitory neurons. Neurogranin is a broad marker for pyramidal neurons (Higo et al., 2004; Singec et al., 2004; Figure 2A) and SMI-32 is a marker for a putative subset of pyramidal neurons that have long-range projections (Voelker et al., 2004; Figure 2A). 73.4% of neurogranin+ pyramidal neurons express D5Rs (Figure 2B). D5R expression was significantly greater among the subset of putative long-range projecting pyramidal (SMI-32+) neurons (93.0%, *p* < 1 × 10^−10^).

We observed a much lower fraction of D5R-expressing cells among inhibitory neurons. D5R expression was examined across four subtypes of inhibitory neuron: parvalbumin+, calretinin+, calbindin+ and somatostatin+ (Figure 2C). Overall, D5Rs were expressed by 52.0% of the four subtypes (Figure 2D). However, the rate of expression differed among those subtypes. Among the parvalbumin+, calbindin+ and calretinin+ inhibitory interneuron subtypes, D5Rs were least prevalent on calretinin+ neurons. Only 40.1% of calretinin+ neurons were also D5R+. This proportion was significantly lower than the proportion of either parvalbumin+ (67.4%, *p* < 1 × 10^−10^) or calbindin+ (61.1%, *p* = 1.22 × 10^−7^) neurons expressing D5Rs.

We next compared the expression of D5Rs between the two most functionally distinct populations, namely the putative projection pyramidal neurons (SMI-32) and inhibitory neuron classes. We found significantly higher proportions of putative projection neurons expressed D5Rs when compared to any class of inhibitory interneuron (parvalbumin *p* < 1 × 10^−10^, calbindin *p* < 1 × 10^−10^, calretinin *p* < 1 × 10^−10^, somatostatin *p* = 5.29 × 10^−5^). D5R is also expressed on proportionally fewer inhibitory interneurons than pyramidal neurons overall, and this difference reached statistical significance in the case of calretinin+ neurons (*p* < 1 × 10^−10^). There was no significant difference in proportion of D5R+ neurogranin+ neurons compared to parvalbumin+ (*p* = 0.14), calbindin+ (*p* = 0.15), or somatostatin+ (*p* = 0.51, Fisher Exact test) neurons. It should be noted that somatostatin+ neurons were extremely rare in the FEF. We found 2.01 somatostatin+ neurons per mm^2^, which is similar to Hendry et al.’s (1984) finding of 2.7–3.2 somatostatin+ neurons per mm^2^ in areas 3b and 4 of macaque cortex. Therefore the calculated fraction of somatostatin+ neurons co-expressing D5Rs was based on a relatively small number of neurons.

In summary, D5R expression varied across cell type, with putative long-range projecting pyramidal neurons disproportionately expressing D5Rs, compared to inhibitory neurons and the neurogranin+ pyramidal neurons. Of the inhibitory neuron classes, D5Rs were most sparsely represented on calretinin+ neurons: fewer than half of calretinin+ inhibitory neurons expressed D5Rs.

### Variation in D5R Expression across Layers and Cell Types

**Figure 3:**
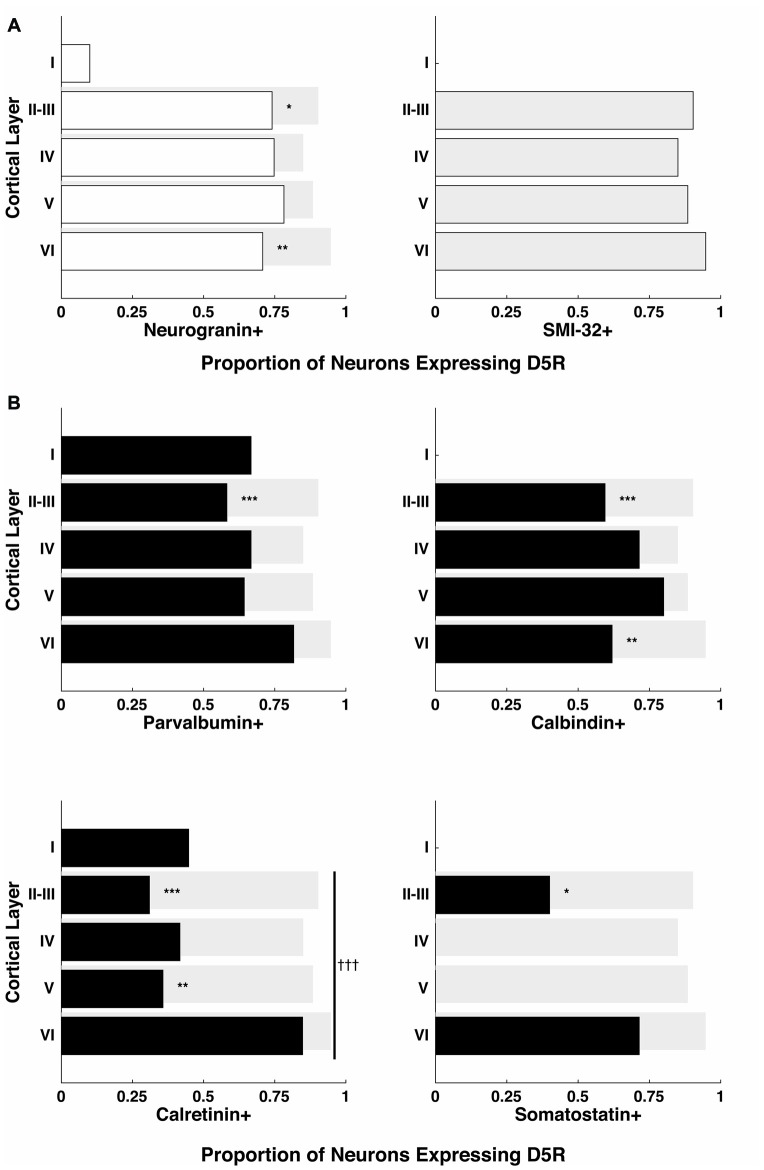
Proportion of D5Rs on different cell types across cortical layers. **(A)** Neurogranin+ pyramidal neurons and SMI-32+ putative long-range projection neurons. **(B)** Parvalbumin+, calbindin+, calretinin+ and somatostatin+ inhibitory interneurons. Proportions are broken down by cortical layer (I, II–III, IV, V and VI) and compared to SMI-32+ D5R+ proportions (light gray bars). *, ** and *** indicate significance at the levels of *p* ≤ 0.05, 0.01 and 0.001 (Bonferonni-adjusted values), respectively. Differences in the proportion of D5R+ neurons across layers were also calculated individually for each cell type. Only calretinin+ neurons exhibited a significant difference in D5R+ proportions across layers: indicated with a vertical line spanning layers II-VI and significance at the level of *p* ≤ 0.001 (Bonferonni-adjusted value) is indicated by ^†††^.

In order to test whether the differential distribution of D5Rs across cell types depended on cortical laminae, we quantified D5R+ expression among pyramidal neurons for cortical layers I–VI (Figure 3A). Because of the scarcity of neurons in layer I, we chose to exclude it from the cross-laminar comparison. Across layers II–VI, the proportion of D5R+ pyramidal neurons (neurogranin+ or SMI-32+) was not significantly different (*p* = 0.35, *p* = 0.99 respectively). Given that the proportion of D5R+ long-range projection (SMI-32+) neurons was significantly higher than the proportion of D5R+ neurogranin+ pyramidal neurons (Figure 2B), we next tested whether this difference was present across all laminae, or restricted to particular layers. We observed the former result. The greater expression of D5Rs on putative long-range projecting neurons compared to neurogranin+ pyramidal neurons was only significant in layers II–III (*p* = 4.95e^−4^) and in layer VI (*p* = 1.02e^−4^; Figure 3A).

Next, we also examined whether the proportion of D5R-expressing inhibitory interneurons varied significantly across cortical laminae. We found no significant difference in the proportion of D5R expression among parvalbumin+ (*p* = 0.6) or calbindin+ (*p* = 0.93) neurons across layers II–VI. However, we observed a significant difference in the proportion of D5R expression among calretinin+ neurons (Figure 3B, bottom left panel, *p* = 6.6 × 10^−4^). For calretinin+ neurons, there D5R expression was higher in layer VI than in other layers. As mentioned above, somatostatin+ neurons were extremely sparse within the FEF. However, we observed that D5R expression among these neurons existed exclusively in layers II–III or layer VI.

Lastly, as with the comparison of SMI-32+ and neurogranin+ neurons, we examined whether the proportionally higher expression of D5R on SMI-32+ neurons, compared to inhibitory neurons, was present across all laminae or restricted to particular layers. For each of the inhibitory interneuron classes, we found the difference was largely restricted to layers II–III (Figure 3B). In each case, there was significantly lower expression of D5R in this layer (parvalbumin+ *p* = 2.84 × 10^−7^, calbindin+ *p* = 2.73 × 10^−7^, calretinin+ *p* < 1 × 10^−10^, somatostatin+ *p* = 8.26 × 10^−4^). The difference in layers II–III was also evident when comparing D5R expression between inhibitory neurons and neurogranin+ pyramidal neurons, with the exception that the difference in somatostatin proportions did not reach significance (parvalbumin+ *p* = 4.04 × 10^−4^, calbindin+ *p* = 2.64 × 10^−4^, calretinin+ *p* < 1 × 10^−10^, somatostatin+ *p* = 0.08). In addition to the robust differences in D5R expression in layers II–III, we found lower D5R expression among layer VI calbindin+ neurons (*p* = 4.14 × 10^−5^) and among layer V calretinin+ neurons (*p* = 2.07 × 10^−7^), when compared to putative long-range projection neurons in the same layers.

In summary, we found that D5Rs are not uniformly distributed across neuronal cell types and layers. There is disproportionately higher D5R expression on putative long-range projecting neurons than neurogranin+ pyramidal neurons specifically in layers II–III and layer VI. At the same time, there is disproportionately lower D5R expression on all classes of inhibitory interneuron types compared to putative long-range projecting neurons in layer II–III. For calretinin+ neurons specifically, the proportion of neurons expressing D5Rs varies significantly across layers, with the highest proportion in layer VI.

### Relative Fractions of D5R+ Cell Types Vary across Layers

**Figure 4:**
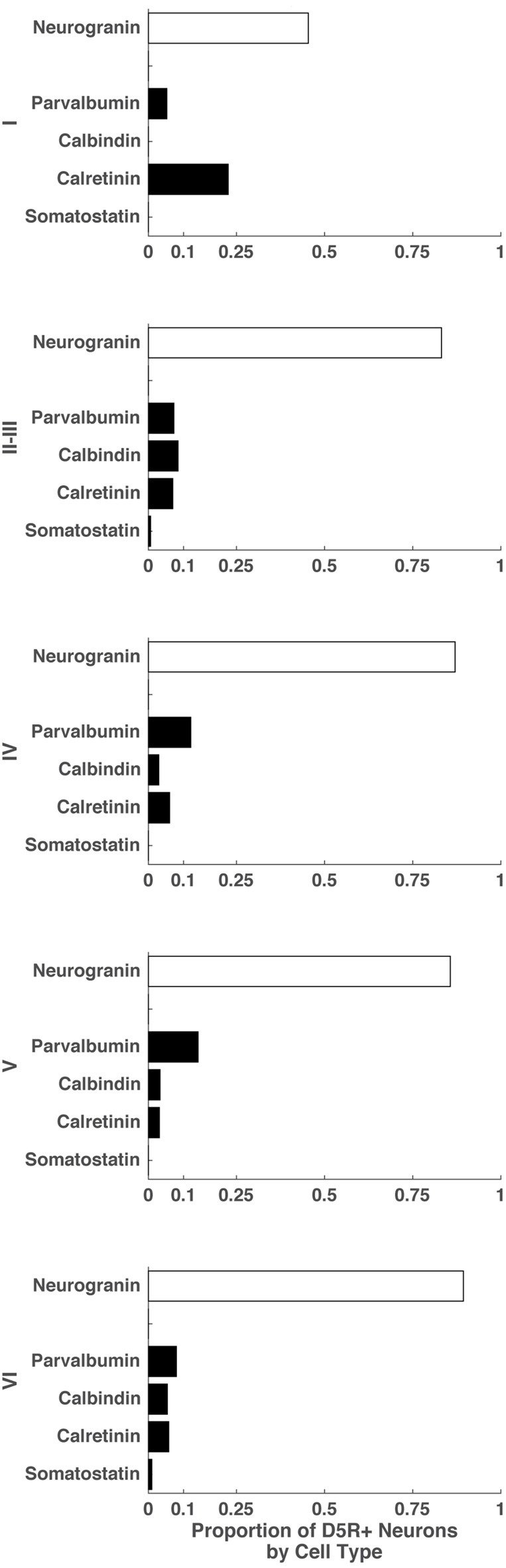
Proportion of D5R+ neurons by cell type for different cortical layers. For cortical layers I, II–III, IV, V and VI: proportion of D5R+ cells that express a given cell type marker (SMI-32, neurogranin, parvalbumin, calretinin, somatostatin).

Finally, we examined the proportion of D5R+ cells that expressed markers for different cell types: indicating the balance of excitatory pyramidal neurons and inhibitory neurons among all D5R+ neurons in individual layers. To do this, we quantified the number of D5R+ neurons in each layer and also the number of D5R+ neurons that co-expressed specific cell type markers. Because we could not stain for all cell types on the same section, our proportions will be approximate and not necessarily sum to 1. Across all layers, the predominant neuron subtype expressing D5Rs were pyramidal neurons (Figure 4). In layer I, the most prominent inhibitory neuron subtype expressing D5Rs were calretinin+ neurons (0.23). There were no calbindin+ or somatostatin+ neurons expressing D5R in this layer. In layer II–III, we saw very similar fractions of D5R+ parvalbumin+, calbindin+ and calretinin+ neurons (0.07, 0.08, 0.07 respectively); though there was a slightly higher proportion of D5R+ calbindin+ neurons. In the remaining layers (IV, V and VI), the predominant inhibitory neuron class expressing D5Rs was parvalbumin+ (layer IV: 0.12, layer V: 0.14, layer VI: 0.08). For most layers, the proportions of D5R+ cell types summed to approximately 1 (Layer II–III: 1.06, Layer IV: 1.08, Layer V: 1.06, Layer VI: 1.09), indicating that our cell type staining encompassed the majority of neuron classes that express D5Rs in these layers. Layer I D5R+ cell type fractions did not sum to approximately 1 (~0.73), indicating the existence of another D5R+ cell type in this layer. In summary, not only does the expression of D5R vary across cell types and layers (Figure 3), but the relative proportions of D5R+ neurons also varies across layers (Figure 4). Across all layers, the majority of D5R+ neurons are pyramidal neurons and among inhibitory neurons, D5R+ calretinin+ neurons are more prevalent in layer I and D5R+ parvalbumin+ neurons are more prevalent in layers IV and V.

## Discussion

### Long-Range Projecting Pyramidal Neurons Disproportionately Express D5Rs

We found that a majority of pyramidal neurons express D5Rs and that pyramidal neurons are also the predominant cell type expressing D5Rs. Previous findings (Bergson et al., 1995a,b; Ciliax et al., 2000) identified D5Rs as primarily being expressed on pyramidal neurons in macaque, which were identified morphologically. We used two different pyramidal-neuron specific markers to show that D5Rs are indeed primarily expressed on pyramidal neurons. Moreover, we found that they are disproportionately expressed on putative long-range projection neurons.

The effect of D5R activation on excitability is complex (Arnsten, 2015; Ledonne and Mercuri, 2017), but in general activation of D5Rs will, through second messengers, cause an increase in intracellular Ca^2 +^ and an overall increase in excitability in neurons (Wang and O’Donnell, 2001; Tseng and O’Donnell, 2004; Chen et al., 2007; Yi et al., 2013). Therefore the high proportion of putative long-range projecting neurons expressing D5Rs means they are more likely to increase their excitability in response to dopaminergic input. Long-range projecting neurons in the FEF have different synaptic targets depending on their layer. Layer II–III pyramidal neurons in the FEF project to other regions of cortex, including both dorsal and ventral areas of extrastriate visual cortex (Schall et al., 1995; Stanton et al., 1995; Anderson et al., 2011). FEF pyramidal projections to visual cortex are thought to shape attention-related modulation of visual activity (Moore and Armstrong, 2003; Ekstrom et al., 2008; Zhou and Desimone, 2011; Gregoriou et al., 2012). Therefore activation of D5Rs by dopamine release in layer II–III will cause an increase in excitability of the majority of long-range projecting FEF neurons, such as those which target visual cortex. This is particularly relevant given the known role of dopamine in attention (Arnsten, 2011; Del Campo et al., 2011; Noudoost and Moore, 2011b; Ranganath and Jacob, 2016). Noudoost and Moore (2011a) showed that pharmacological manipulation of D1/5Rs in the FEF caused changes in visually driven activity within area V4 that was similar to attentional modulation. Activation of D5Rs, should they cause an increase in neuronal excitability, is one mechanism through which such changes could be effected. However, application of D1/5R agonists does not necessarily cause an increase in neuronal responses, and can frequently cause a decrease in activity. Indeed Noudoost and Moore (2011a)’s application of a D1/5R antagonist in the FEF caused increases in activity in area V4. The development of new drugs that can isolate activation/inhibition of either D1Rs of D5Rs will help explain how these receptors cause changes in excitability of specific cell types.

Many models of prefrontal cortical functions such as attention and working memory rely on persistent activity that is generated by recurrent connectivity of pyramidal neurons (Wang, 1999; Durstewitz et al., 2000; Deco and Rolls, 2003; Riley et al., 2017). Increased excitability of long-range projecting pyramidal neurons via D5R activation would facilitate maintenance of signals through long-range recurrent connections. Notably, layer V neurons, which project to brainstem oculomotor structures (Schnyder et al., 1985; Borra et al., 2015), did not exhibit a disproportionate expression of D5Rs. Therefore, dopamine presence in the region is more likely to affect layer II–III pyramidal neurons, which project to visual cortices, than layer V pyramidal neurons which project to brainstem oculomotor structures.

We also observed a significant difference in the proportion of D5R+ putative long-range projection neurons compared to neurogranin+ pyramidal neurons in layer VI. Layer VI pyramidal neurons, canonically, project to either the thalamus or to other cortical regions as well (Briggs, 2010). One major thalamic target for layer VI FEF neurons is the mediodorsal thalamic nucleus (MD; Xiao et al., 2009). The majority of these putative projection cells will also be influenced through D5R-dependent mechanisms.

### D5Rs Are Less Prevalent on Inhibitory Interneurons

Our study also quantified D5R expression on both inhibitory neurons and pyramidal neurons so that they can be compared directly. We show that D5R expression is less prevalent among inhibitory neurons compared to neurogranin+ pyramidal neurons, and especially when compared to putative long-range projection neurons. Indeed, we found that a significantly lower proportion of all classes of interneurons express D5R in layer II–III than putative long-range projection neurons. Given that the proportion of putative long range projection neurons that express D5Rs is also significantly higher than the proportion of D5R+ pyramidal neurons overall in layer II–III, dopamine activation of D5Rs is more likely to alter the excitability of long-range projection neurons in layer II–III of the FEF and less likely to alter the excitability of inhibitory interneurons in the same layer. Lower excitability of inhibitory neurons overall could result in less inhibition in layer II–III which could also facilitate persistent activity through pyramidal neurons. However, models of persistent activity computations in the PFC propose parvalbumin+, calbindin+ and calretinin+ neurons in layer II–III play unique roles in shaping the spatial tuning of persistently active pyramidal neurons (Wang et al., 2004; Konstantoudaki et al., 2014; Murray et al., 2015). This suggests a more nuanced role for D5Rs in the PFC microcircuit, which will benefit from further investigation in future studies.

### D5R Expression on Neurons Varies across Cortical Layers

Overall, our findings match previously described observations of D5R expression patterns across different cortical layers. We found that among all neuronal types the peak proportion of D5R positive neurons was in layer V, a finding that confirms at the protein level previous evidence at the transcriptional level (Lidow et al., 1998). We also see higher expression of D5Rs across layers II–VI than layer I, which matches a previous qualitative report of D5Rs being uniformly distributed across these layers in macaque PFC (Ciliax et al., 2000). Our results also extend our understanding of D5R expression across cortical layers. We found differences in the proportion of different cell types expressing D5R in deeper layers. The most striking of these results was the significant difference in the proportion of D5R+ calretinin+ neurons across layers II–III, IV, V and VI. The proportion of calretinin+ neurons that express D5R was clearly much higher in layer VI than other layers. Calretinin+ neurons themselves are actually sparsest in deeper layers of cortex (Condé et al., 1994; Dzaja et al., 2014). This means that calretinin+ neurons’ inhibition of other interneurons will be more strongly modulated through D5R receptors in deeper layers, where calretinin+ neurons are already rare, than in any other layer including layers I and II–III, where the calretinin+ neurons are densest. Calretinin+ neurons primarily inhibit other interneurons (Meskenaite, 1997; Gonchar and Burkhalter, 1999; Staiger et al., 2004; Wang et al., 2004). Therefore D5R activation of calretinin+ neurons in layer VI would lead to an increase in their excitability and therefore a more powerful inhibition of other inhibitory interneurons. This in turn would facilitate cortico-cortical and cortico-thalamic signaling.

### Potential Limitations

In this study, we identified neurons as being D5R-positive or D5R-negative solely on the basis of D5R presence or absence on neuronal cell bodies. We did not explore the expression of D5Rs on neuronal processes, so it is possible our quantification of D5R expression underestimates the true number of neurons expressing this receptor. However, several previous findings lead us to have confidence in the accuracy of whole cell counts relative to neuropil. Bergson et al. (1995b) and Ciliax et al. (2000) found that D5Rs were primarily expressed on neuronal somas and proximal dendrites in the cerebral cortex, and more prevalent in dendritic shafts than dendritic spines. Also, Glausier et al. (2009), who primarily quantified D5R expression on the neuropil of calretinin+ and parvalbumin+ neurons in layers I, III and V of macaque dorsolateral PFC, found that in layer III 15% of calretinin+ dendritic profiles and 4% of parvalbumin+ dendritic profiles expressed D5R. Although there is not necessarily a correspondence between dendritic D5R expression and somatic D5R expression, the fact that these values are lower than our somatic counts is consistent with previous studies (Bergson et al., 1995b; Ciliax et al., 2000) report that D5Rs are more prevalent on neuronal somas than in the neuropil.

### Future Directions

Although for this study we have focused exclusively on D5Rs, the most abundant member of the D1-like dopamine receptor family (Bergson et al., 1995b; Bordelon-Glausier et al., 2008), and the member most strongly activated by dopamine (Sunahara et al., 1991; Weinshank et al., 1991; Tiberi and Caron, 1994); it will also be important to determine whether there are any difference in cell type- and layer-specific expression of D5Rs compared with D1Rs. Bordelon-Glausier et al. (2008) showed that D5R and D1R have broadly overlapping expression in layers I, III and V of macaque dlPFC, but more work needs to be done to elucidate the D1-like receptor response circuit to dopamine in the PFC.

## Author Contributions

AM and TM contributed to the conception and design of the study. AM and SBS performed the immunofluorescence experiments. AM performed the microscopy and statistical analysis and wrote the first draft of the manuscript. All authors contributed to manuscript revision, read and approved the submitted version.

## Conflict of Interest Statement

The authors declare that the research was conducted in the absence of any commercial or financial relationships that could be construed as a potential conflict of interest. The handling editor is currently co-hosting a Research Topic with one of the reviewers AD, and confirms the absence of any other collaboration.

## Supplementary Material

FIGURE S1 D5 class of dopamine receptor (D5R) antibody specificity controls. **(A)** Western blot of D5R antibody. D5Rs are present on white blood cells, which are found in the serum of a routine blood draw. We performed a western blot using three volumes of the extracted macaque serum and consistently found the Almone D5R antibody stained one major band at approximately 44 kD. Macaque D5R is theoretically predicted to have a molecular weight of 52.9 kDa. Proteins often do not run exactly at their predicted weight due to post-translational modification such as phosphorylation or glycosylation, which can cause different mobilities on SDS-PAGE. Proteins can also appear at lower positions than expected due to partial digestion by enzymes. **(B)** Preadsoprtion control of D5R antibody. Two neighboring sections of FEF were stained with the Alomone D5R antibody. The left section was stained using the described methods. The right section was stained after preadsorbing the same volume of antibody (500 μl at 1:200) with D5R antigen supplied by Alomone (40 μg) overnight at 4°C. After preadsorbption no staining is visible on the section—indicating a lack of non-specific binding. Scale bars = 200 μm.
