# Supplementary figures and images for: Differential Expression of Dopamine D5 Receptors across Neuronal Subtypes in Macaque Frontal Eye Field
Source: Front Neural Circuits. 2018 Feb 12;12:12. doi: 10.3389/fncir.2018.00012 (PMC5816032; doi:10.3389/fncir.2018.00012)

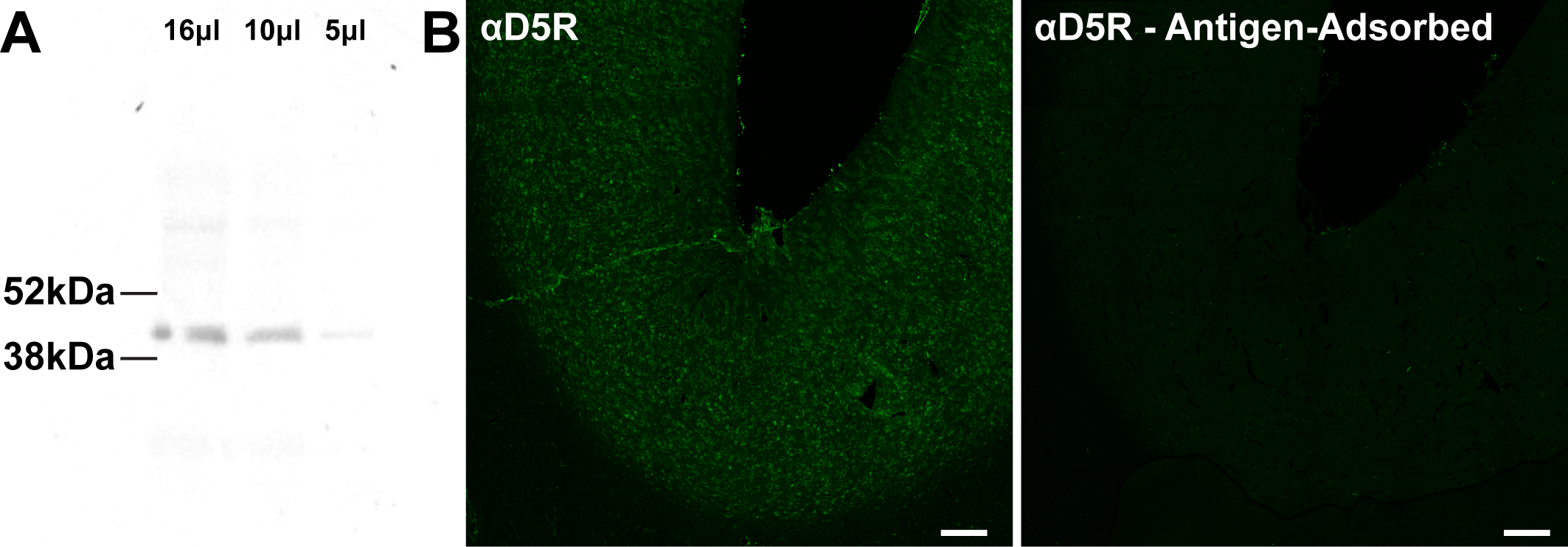

Supplement: FIGURE S1 — D5 class of dopamine receptor (D5R) antibody specificity controls. (A) Western blot of D5R antibody. D5Rs are present on white blood cells, which are found in the serum of a routine blood draw. We performed a western blot using three volumes of the extracted macaque serum and consistently found the Almone D5R antibody stained one major band at approximately 44 kD. Macaque D5R is theoretically predicted to have a molecular weight of 52.9 kDa. Proteins often do not run exactly at their predicted weight due to post-translational modification such as phosphorylation or glycosylation, which can cause different mobilities on SDS-PAGE. Proteins can also appear at lower positions than expected due to partial digestion by enzymes. (B) Preadsoprtion control of D5R antibody. Two neighboring sections of FEF were stained with the Alomone D5R antibody. The left section was stained using the described methods. The right section was stained after preadsorbing the same volume of antibody (500 μl at 1:200) with D5R antigen supplied by Alomone (40 μg) overnight at 4°C. After preadsorbption no staining is visible on the section—indicating a lack of non-specific binding. Scale bars = 200 μm. [file Image_1.tif]
